# Distinct microRNA Expression Profile in Prostate Cancer Patients with Early Clinical Failure and the Impact of *let-7* as Prognostic Marker in High-Risk Prostate Cancer

**DOI:** 10.1371/journal.pone.0065064

**Published:** 2013-06-14

**Authors:** Maria Schubert, Martin Spahn, Susanne Kneitz, Claus Jürgen Scholz, Steven Joniau, Philipp Stroebel, Hubertus Riedmiller, Burkhard Kneitz

**Affiliations:** 1 Department of Urology and Pediatric Urology, Comprehensive Cancer Center (CCC) Mainfranken, University Hospital, Würzburg, Germany; 2 Department of Urology, University Hospital, Bern, Switzerland; 3 Department of Physiologic Chemistry, University of Würzburg, Würzburg, Germany; 4 Microarray Applications, University of Würzburg, Würzburg, Germany; 5 Department of Urology, University Hospital, Leuven, Belgium; 6 Department of Pathology, University Hospital, Göttingen, Germany; Innsbruck Medical University, Austria

## Abstract

**Background:**

The identification of additional prognostic markers to improve risk stratification and to avoid overtreatment is one of the most urgent clinical needs in prostate cancer (PCa). MicroRNAs, being important regulators of gene expression, are promising biomarkers in various cancer entities, though the impact as prognostic predictors in PCa is poorly understood. The aim of this study was to identify specific miRNAs as potential prognostic markers in high-risk PCa and to validate their clinical impact.

**Methodology and Principal Findings:**

We performed miRNA-microarray analysis in a high-risk PCa study group selected by their clinical outcome (clinical progression free survival (CPFS) vs. clinical failure (CF)). We identified seven candidate miRNAs (*let-7a/b/c, miR-515-3p/5p, -181b, -146b*, and *-361*) that showed differential expression between both groups. Further qRT-PCR analysis revealed down-regulation of members of the *let-7* family in the majority of a large, well-characterized high-risk PCa cohort (n = 98). Expression of *let-7a*/*b*/and *-c* was correlated to clinical outcome parameters of this group. While *let-7a* showed no association or correlation with clinical relevant data, *let-7b* and *let-7c* were associated with CF in PCa patients and functioned partially as independent prognostic marker. Validation of the data using an independent high-risk study cohort revealed that *let-7b,* but not *let-7c,* has impact as an independent prognostic marker for BCR and CF. Furthermore, we identified *HMGA1*, a non-histone protein, as a new target of *let-7b* and found correlation of *let-7b* down-regulation with HMGA1 over-expression in primary PCa samples.

**Conclusion:**

Our findings define a distinct miRNA expression profile in PCa cases with early CF and identified *let-7b* as prognostic biomarker in high-risk PCa. This study highlights the importance of *let-7b* as tumor suppressor miRNA in high-risk PCa and presents a basis to improve individual therapy for high-risk PCa patients.

## Introduction

Prostate cancer (PCa) is the most common malignancy among men in Europe, with an estimated incidence of 345,900 in 2006 [1]. The natural course of the disease is heterogeneous, varying from indolent to highly aggressive cancer that metastasizes at early stage causing pain and untimely death. Current risk stratifications like low−/intermediate−/and high-risk PCa alone are insufficient to predict clinical outcome. Even men with high-risk PCa (PSA >20 ng/ml and/or biopsy Gleason Score ≥8 and/or clinical stage ≥ T3) represent a heterogeneous group of patients. Even though characterized as a group that has poorest clinical outcome among all risk groups, only up to 30% develop metastases and die due to their disease [2]–[4]. Therefore, new prognostic biomarkers are urgently needed to better sub-stratify risk groups, identify the lethal disease, eventually avoid overtreatment, and improve individual therapy. The identification of prognostic markers, especially for the lethal disease, is hardly possible in an unselected PCa collective. Although high-risk PCa cohorts now show much better than expected outcomes, they still represent an ideal group to identify factors specifically correlated with the lethal disease.

There is growing evidence that microRNAs (miRNA) are suitable candidates for the development of such biomarkers. MiRNAs are small non-coding RNA strands that regulate expression of genes at the post-transcriptional and the translational level. Individual miRs have been characterized either as tumor suppressors or oncogenes (oncomiRs) [5].

Several reports describe PCa-specific miRNA expression signatures, however the kind of regulated miRNAs is diverse. Agreement exists among these studies in that the majority of miRNAs are down-regulated in the PCa samples [6]–[10]. Although a correlation to tumor stage and grade was described for several miRs their relevance as prognostic markers to predict hard clinical endpoints, like clinical failure or cancer-related death, remains limited [8], [11]–[14]. However, there are promising approaches to detect coherence between altered expression of specific miRNAs and progression of the disease. Larne and coworkers recently identified a miRNA-based multimarker model as prognostic tool for progression in PCa [15]. Our working group previously described *miR-221* to be a prognostic marker for disease recurrence in high-risk PCa [16].

Some of the most frequently mentioned miRs that show down-regulation in PCa are members of the *let-7* family [6], [9], [17], [18]. This family consists of several members, whose diversity is distinct by isoforms (*let-*7*a-g,-i, miR-98*). The expression of some *let-7* members was shown to be down-regulated in various other cancer entities as well, such as breast, ovarian, and lung cancer [19]–[21]. Known relevant targets of *let-7* are oncogenes like *Ras*, *cmyc*, *EZH2* and *HMGA2* [17], [22]–[24], indicating a tumor-suppressive function of *let-7* by regulating oncogenes specifically involved in growth and self-renewing capacity of PCa cells. More recently it was shown that PCa stem cells are characterized by down-regulation of *let-7* family members and that *let-7* is critically involved in tumorigenicity by controlling androgen receptor signaling, proliferation and differentiation [25]–[27]. However, a role of *let-7* family members as prognostic markers in PCa has not been described up to now.

The aim of this study was to identify miRNAs differentially expressed in high-risk PCa with diverse clinical outcome. We detected a pattern consisting of 7 miRNAs associated with early clinical recurrence and identified *let-7* family members to be progressively down-regulated in aggressive tumors. In a large high-risk PCa cohort we confirmed these results and demonstrated that down-regulation of *let-*7*b* is correlated with biochemical recurrence and clinical failure. Furthermore, we confirmed *let-7b* as independent prognostic marker in high-risk cancer in an independent validation cohort. In addition we showed that expression of *HMGA1*, a non-histone protein, is regulated by *let-7b* by binding to the 5′UTR of *HMGA1*. Our results demonstrate that high-risk PCa is characterized by a specific miRNA profile and that individual *let-7* family members are promising prognostic markers in this patient group.

## Materials and Methods

### Patient Cohorts

We worked with three diverse PCa collectives for our analyses:

Cohort A consists of 98 formalin fixed and paraffin embedded (FFPE) tissue specimen of a well-characterized group of high-risk PCa patients [16]. See table 1 for clinic-pathological data. Tissue samples and clinical data were utilized for microarray and qRT-PCR analyses on microRNAs as well as subsequent correlation and association studies.

**Table 1:** Clinico-pathological features of both high-risk PCa groups (cohort A – test group and cohort B – validation group).

	Cohort A (n = 98)	Cohort B (n = 92)
Clinic-pathological features	n (%)	n (%)
age, years (range)	66 (47–78)	64 (41–76)
follow-up, months (st.dev)	78 (±38)	111 (±45)
pre-operative PSA (ng/ml)		
mean (range)	48 (20–156)	22 (1–95)
Gleason Score		
≤6	3 (3.1%)	26 (28.3%)
7	39 (39.8%)	46 (50%)
8	15 (15.3%)	12 (13%)
9	29 (29.6%)	8 (8.7%)
10	12 (12.2%)	0 (0%)
pathological tumor stage		
pT2	16 (16.3%)	29 (31.5%)
pT3a	23 (23.5%)	36 (39.1%)
pT3b	42 (42.9%)	21 (22.9%)
pT4	17 (17.3%)	6 (6.5%)
lymph node		
positive	34 (34.7%)	10 (10.9%)
negative	64 (65.3%)	82 (89.1%)
clinical failure		
yes	18 (18.4%)	14 (15.2%)
no	80 (81.6%)	78 (84.8%)

Cohort B consists of 92 FFPE samples from RP. Tissue specimen and clinic-pathological data were obtained from the Department of Urology at the University Hospital Leuven, Belgium (Table 1). This group served as validation collective to confirm the role of *let-7b* as prognostic marker.

Preoperative staging in cohort A and B included DRE, an abdominopelvic-computed tomography (CT) scan and a bone scan. Neoadjuvant hormonal treatment, radiation or chemotherapy was an exclusion criterion. Lymph node metastasis and prostate specimens (whole mount sections, 4 mm intervals) were staged and graded according to the 2002 TNM classification and the Gleason grading system as previously described [16]. Follow-up was performed every 3 months for the first 2 years after surgery, every 6 months in the following 3 years, and annually thereafter. Biochemical relapse (BCR) was defined as PSA ≥0.2 ng/ml on 2 consecutive follow-up visits. Clinical failure was declared when either local or distant metastases were histologically proven or confirmed by CT or bone scan. Overall survival (OS) was defined as time from RP to death attributed to PCa or complications of the disease.

Benign prostatic hyperplasia (BPH) samples were derived from prostate adenomectomy specimens. Samples were paraffin-embedded as well; regions with >80% adenoid tissue were used. All patients had normal PSA levels before surgery and carcinoma was excluded by histopathology.

Cohort C consists of 21 cancer samples obtained from the Department of Urology at the University Hospital Wuerzburg, Germany. Pairs of fresh frozen PCa tissue and adjacent benign tissue, were used for mRNA and miRNA isolation. This group contains patients with unselected histo-pathological PCa specimen (high-, intermediate-, and low risk cancer). Since mRNA isolation of fresh frozen tissue specimen is more effective compared to isolation on FFPE samples we worked with this cohort for correlation studies on expression of *HMGA1* and *let-7b*. For this analysis clinic-pathological data were irrelevant and therefore not shown. Histological evaluation was performed by a Senior pathologist (P.S.). Cancerous samples containing at least 80% malignant cells were used for further analyses. Areas with at least 80% ducts and no cancerous cells were selected for adjacent benign tissue.

The studies were approved by the local ethics committee of the medical faculty of the University of Wuerzburg, Germany (no. 59/04) and the Catholic University Leuven, Belgium (UZ Leuven Study number S54424; Belgian Study number B322201214832); all patients provided written informed consent.

### Microarray Analysis

To screen for candidate miRs, being correlated with poor outcome 6 BPH tissues and 13 high-risk PCa specimens were hybridized to microarrays (cohort A). High-risk PCa specimens were subdivided into two groups (group 1: CPFS (n = 7); group 2: CF (n = 6) (see Table 2 for details). A set of 668 miRs (Probe Set 1564V2 miRVana Applied Biosysems) was spotted on Nexterion™ HiSense E microarray slides in quadruplicates. We used the Pure-Link FFPE Total RNA Isolation Kit and the RiboMinus Concentration Module (Invitrogen) for RNA purification. Slide processing was performed according to the Applied Biosystems *miR*Vana™ manuals. The microarray data is available under GEO accession number GSE18671.

**Table 2:** Clinic-pathological features of 13 high-risk PCa patients (cohort A) separated by clinical outcome (group 1: high-risk PCa+CPFS; group 2: high-risk PCa+CF).

Clinic-pathol. features	Group 1	Group 2
	High-risk PCa+CPFS (n = 7)	High-risk PCa+CF (n = 6)
age, years (range)	64 (52–74)	66 (57–71)
follow-up, months (st.dev)	106 (±34.1)	72.6 (±25.9)
pre-operative PSA (ng/ml)	52.27 (21–79)	63.96 (24–125)
Gleason Score		
6	3	0
7	4	0
8	0	0
9	0	1
10	0	5
pathol. tumor stage		
pT2b	1	0
pT3a	1	0
pT3b	3	1
pT4	2	5
surgical margin status		
pos.	6	6
neg.	1	0
biochemical relapse		
yes	3	6
no	4	0
clinical failure		
yes	0	6
no	7	0
cancer-related death		
yes	0	3
no	7	3

### RNA Extraction and Reverse Transcription

Total RNA extraction from paraffin-embedded or frozen tissue samples and PCa cell lines were performed using the Recover all Total Nucleic Acid Isolation Kit and the Total RNA Extraction Kit respectively (Ambion and miRNeasy Mini Kit, Qiagen). Specific cDNA was synthesized from total RNA with stem-loop reverse transcription primers according to the TaqMan® miR assay protocol (PE Applied Biosystems). Unspecific cDNA synthesis was performed with ImProm-II™ Reverse Transcription System (Promega) according to the ABsolute QPCR SYBR Green protocol (Thermo Scientific).

### qRT-PCR

Confirmation of the microarray results on independent samples and on an increased sample size was done using qRT-PCR. M(i)RNA expression in tissue samples and cell lines was quantified with either TaqMan® (miR) or SYBR Green (mRNA) assay kits and the BioRad OPTICON 2, following the manufacturer’s instructions (BioRad). Primers for *let-7a-d, miR-16, -29a, -221, -146b,* and *-181b* were obtained from Applied Biosystems; small nuclear RNA *RNU6B* was applied for normalization. *β-Actin* served as housekeeper for *HMGA1* expression (Applied Biosystems). Relative m(i)RNA-expression was calculated with the comparative ΔC_t_-method (ΔC_t_ sample = C_t_ sample - C_t_ *RNU6B*). The 2^–ΔΔCt^ method was used to assess fold changes in m(i)R-expression between samples and controls. Mean C_t_ was determined from triplicate PCR experiments. Standard deviation was ≤0.4, p values <0.05 were considered significant.

### Cell Culture and Transient Transfection

LNCaP and PC-3 cells were obtained from ATCC, Germany using passages 5 to 35. Cells were grown at 37°C in a humidified incubator at 5% CO_2_. Cells were seeded and simultaneously transfected in 6-well plates at a density of 3.5×10^5^ per well using RPMI 1640 Medium Gibco without Phenolred, containing 10% fetal calf serum (Invitrogen), Glutamax (Invitrogen), NEAA (Gibco), HEPES Buffer solution (PAA), and Sodium pyruvate solution (PAA). Transient transfection was performed using Lipofectamine™ reagent (Invitrogen) and Opti-MEM® (Invitrogen-Gibco) following the supplier’s instructions. Precursor- respectively antigo *let-7b* was applied to induce or suppress *let-7b* expression, pre- and anti-negative *let-7b* (Applied Biosystems) were used for control transfection. Cells were harvested 48 h after transfection.

### Western Blot

Cells were lysed in Cytobuster or Phosphosafe (Novagene) after preparation and determination of equal amounts proteins were separated on SDS-PAGE and later transferred to Nitrocellulose membrane (Bio-Rad). For protein expression of *HMGA1* we used 1 mg/ml goat polyclonal antibodies (*HMGA1a/1b*, Abcam); *β-Actin* (Abcam) served as housekeeper. For visualization we used horseradish peroxidase-coupled secondary antibodies (Abcam and Dako) and the ECL Plus kit (GE Healthcare). For quantification of band intensity we used Image J (http://imagej.nih.gov/ij/).

### Luciferase Assay

The 3′UTR of the human *HMGA1* gene was PCR-amplified using the following primers that contained additional Hind III or Spe I sites: HMGA1Hindfor: 5′-GATC AAGCTTCATATTGTGGTGATGGAG-3′; HMGA1SpeIrev: 5′-CAAT ACTAGT GAACATTTGGCGCTGGTAG-3′. The resulting *HMGA1* PCR fragment containing the two most downstream located *let-7b* complementary sites to *HMGA1* was cloned downstream of the Renilla luciferase stop codon into a luciferase reporter plasmid (pMIR- REPORT-Luciferase, Apllied Biosystems) using the Hind III and Spe I sites. To perform luciferase assays LNCaP cells were seeded in 6-well plates at a density of 3.5×10^5^ per well and incubated for 24 hours. The reporter vector was co-transfected into LNCaP cells with a control non-targeting RNA oligonucleotide or *let-7* precursor miRNA. Transient transfection was performed using Lipofectamine™ reagent (Invitrogen). 48 hours post transfection the luciferase activity was analyzed by Dual-Luciferase® Reporter Assay System (Promega). Renilla activity was used for normalization and as a control for transfection efficiency.

### Statistical and Bioinformatics Analysis

Microarray-Analysis: Spot intensities from scanned slides were quantified using ScanAlyze Software (http://rana.lbl.gov/EisenSoftware.htm). Data were analyzed with different R packages from the Bioconductor project (www.bioconductor.org). Resulting signal intensities were normalized by variance stabilization [28]. Differentially expressed genes were selected from the microarray data by limma (Linear models for microarray Analysis) package [29].

qRT-PCR-Analysis: Samples were compared with a Welch Two Sample t-test. Prior to further analysis of collective A, z-scores of the relative expression values were created. Subsequently, the optimal cut-off point for *let-7* expression was determined by receiver operating characteristic (ROC) curve analysis (R-package ‘pROC’). Based on the resulting threshold the expression values were dichotomized. The associations between expression and clinical recurrence were determined by Cox regression with uni- and multivariate models. The Kaplan-Meier method was used to estimate the survivor function at various time points. Univariate and multivariate hazard ratios and their 95% confidence intervals (CI) were estimated using the Cox proportional hazards model (Bioconductor package ‘survival’). P values <0.05 were considered significant. To test, whether this approach is applicable to an independent test dataset, z-scores were created for collective B. However, for dichotomization the threshold derived from collective A was used. All subsequent steps were performed as described for collective A. For correlation between *HMGA1* and *let-7b* Pearson correlation was calculated.

## Results

### Cancer Specific miRNA Expression Status in High-risk PCa

We performed microarray analysis of miRNA isolated from six paraffin-embedded BPH and 13 PCa samples to screen for candidate miRNAs being associated with the development or progression of high-risk disease. The analyzed PCa cases were part of a well-characterized high-risk collective (cohort A) (Table 1) and were selected based on their clinical outcome. The pre-selected PCa cases were separated into two groups by clinical failure and follow-up without recurrence of the disease (group 1: CPFS vs. group 2: CF). Table 2 shows the cancer characteristics of both groups.

**Figure 1:**
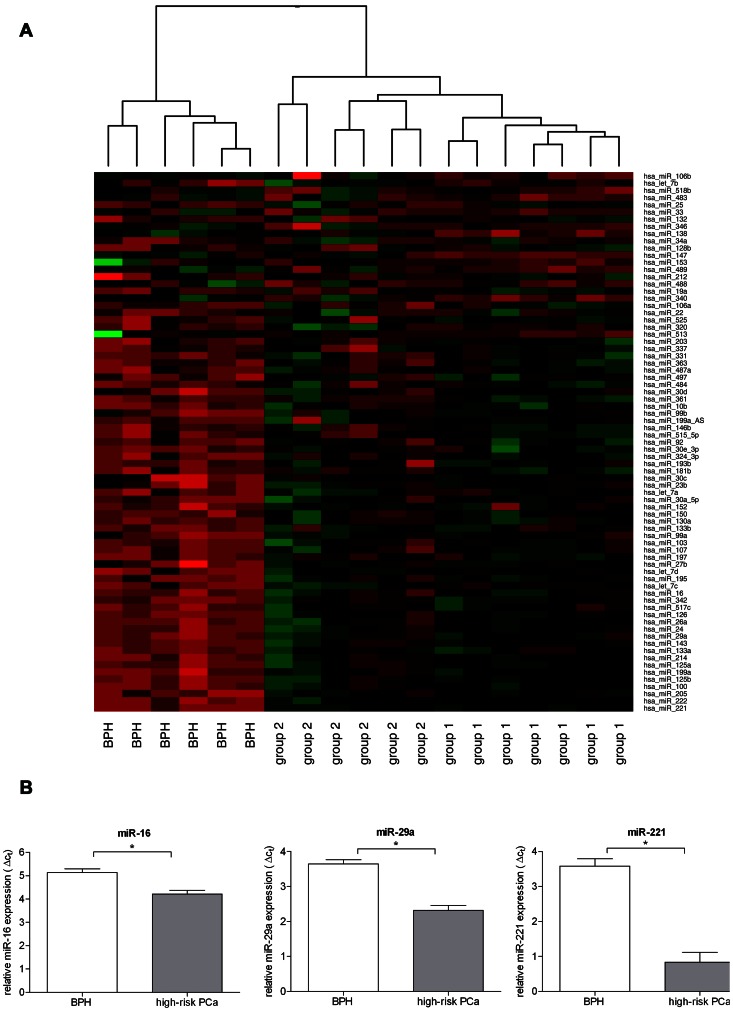
Microarray analysis in high-risk PCa and BPH tissue. A. Microarray profiling of 6 BPH and 13 high-risk PCa tissue specimens (cohort A). The latter were subdivided by clinical outcome: group 1: high-risk PCa+CPFS (n = 7); group 2: high-risk PCa+CF (n = 6). Row-wise scaled heatplot of genes showing a fold change >1.5 and an adjusted p value <0.05. Red indicating high expression, green low expression. Cancerous and non-cancerous prostate tissues are clearly separated. Clustering indicates a distinction between group 1 and 2. B. Technical validation of the microarray data by qRT-PCR. Relative expression of *miR-16*, *-29a*, and *-221* was analyzed in 6 BPH (white) and 13 high-risk PCa samples (grey). MiRNA expression is shown as means of BPH and high-risk PCa expression with error bars for standard deviation. *indicates p<0.01, p values were calculated using the Welch 2 sample t-test.

The goal of this analysis was to screen for a specific miRNA signature of high-risk cancer patients with disease recurrence. Initially we compared miRNA expression in all 13 high-risk PCa cases together to that of the benign disease. Of all differentially expressed miRNAs (expression fold change >1.5 and p value <0.05) the majority of 61 miRs were down-regulated and 21 miRs were up-regulated in the high-risk disease. See Table S1 for listing of all up- and down-regulated miRNAs. Hierarchical clustering, based on the selected 82 differentially expressed miRNAs, generated a tree with clear distinction between the malignant disease and the benign hyperplasia (Fig. 1A). Even though comparison was performed only between the benign and the malignant disease, a sub-grouping of the tumors based on their clinical outcome was evident.

**Figure 2:**
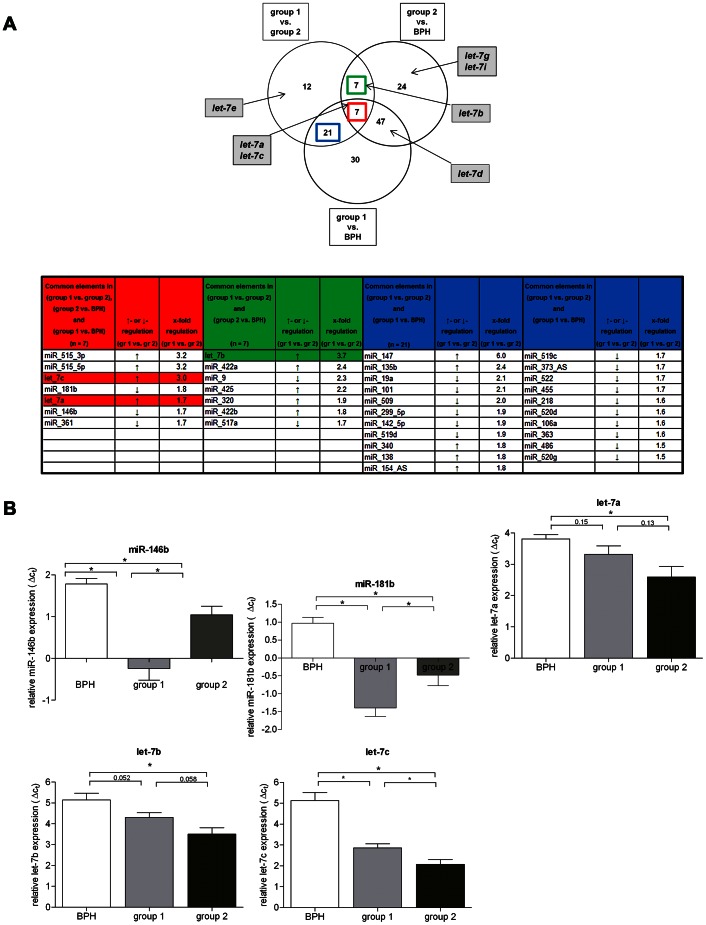
MiRNA expression in high-risk PCa (cohort A) with early clinical failure. A. Venn diagram showing relationships between human miRs that were expressed significantly different (±1.5 fold) in PCa group 1 vs. PCa group 2 vs. BPH (adj p<0.05). Circles include numbers of up- or down-regulated human miRs of each pair-wise comparison. Common miRs between different comparisons are shown in intersections. Grey boxes indicate expression status of the *let-7* family members. The table below lists all human miRs in which expression was significantly different either: between (gr.1 vs. gr.2), (gr.2 vs. BPH) and (gr.1 vs. BPH) (red column); between (gr. 1 vs. gr. 2) and (gr. 2 vs. BPH) (green column); or between (gr. 1 vs. gr. 2) and (gr. 1 vs. BPH) (blue column). All miRNAs are ranked based on the maximum expression fold change. The two columns aside the list of miRNAs indicate an up- or down-regulation in expression (see arrows) and the dimension of expression fold change. B. Validation of the microarray data by qRT-PCR. Relative expression of *miR-146b*, *-181b*, and *let-7a*/*b*/and *-c* was analyzed in 6 BPH (white), 7 high-risk PCa samples with CPFS (group 1) (light grey), and 6 high-risk PCa samples with CF (group 2) (dark grey). MiRNA expression is shown as means of expression in BPH and high-risk tissue, respectively with error bars for standard deviation. *indicates p<0.01, p values were calculated using the Welch 2 sample t-test.

To perform a technical validation of the array results we analyzed the expression of three of the most differentially expressed miRNAs, among others. As shown in Figure 1B and 2B we could confirm the significant down-regulation of *let-7a/b/c, miR-16*, *-29a*, *-146, -181* and *-221* prior seen in the array data by qRT-PCR.

### MiR Expression Profile of High-risk PCa with Diverse Clinical Progression-free Survival

Based on the separation of both risk groups by cluster analysis we hypothesized to find a miRNA profile in PCa cases with progressive disease. Therefore, we analyzed the microarray data with regard to miRs that were differentially expressed in both high-risk groups (fold change >1.5 and P value <0.05). The Venn diagram in Figure 2A and Figure S1 visualize the plotting of all human miRs that were differentially expressed in BPH tissue, in high-risk PCa tissue with CPFS, and in high-risk tissue with CF. In total, 148 miRs were diversely expressed within all three tissue types. The expression of seven miRNAs (namely *let-7a/c, miR-515-3p/5p, -181b, -146b*, and *-361*) were found to be significantly different between all three groups analyzed (BPH, group 1 and 2) indicating a specific miRNA profile for high-risk PCa with progressive disease. 47 miRNAs showed different expression in group 1 vs. group 2; among the top ten most differentially expressed miRNAs we found several members of the *let-7* family (Fig. S1).

Using RT-PCR analysis expression differences between BPH, group 1 and 2 for *let-7a, let-7c*, *miR-146b*, and *-181b* could be confirmed (Fig. 2B). Since expression of *let-7b* and *-d* was shown to discriminate at least two of the three tissue types we included both *let-7* family members in further analyses. As predicted by the array data, the *let-7* family members showed a progressive down-regulation from benign to malignant (group 1) and further to the aggressive disease (group 2), whereas expression of *miR-146b* and *-181b* was lowest in group 1 (Fig. 2B). *Let-7d* expression was hardly detectable by RT-PCR and was, therefore, excluded in further investigations.

### Expression of *let-7a/b/c* is Significantly Down-regulated in High-risk PCa

By microarray and RT-PCR analyses we identified some *let-7* family members (*let-7a-c*) as potential candidate miRs for diagnostic and prognostic markers in high-risk PCa.

**Figure 3:**
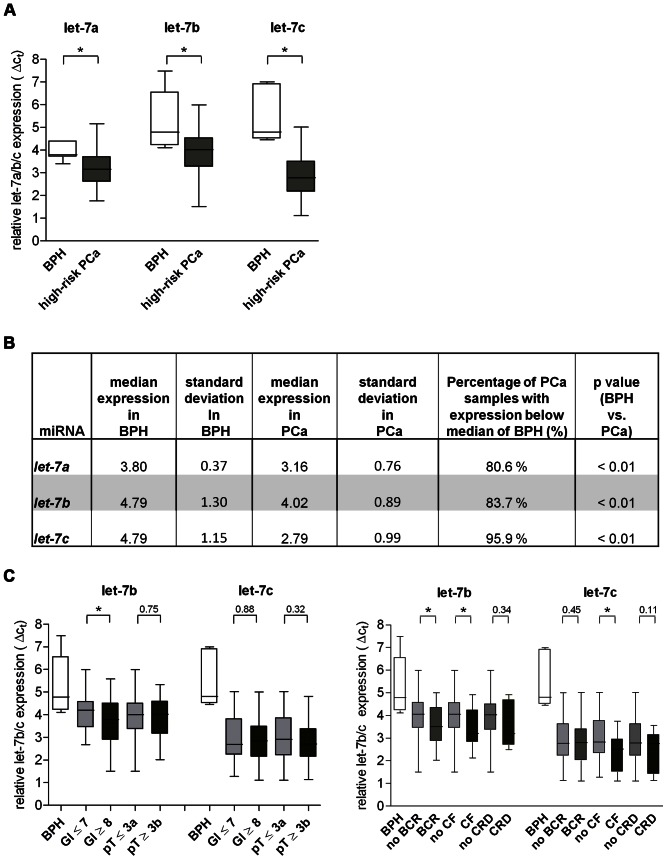
*Let-7a/b/c* expression in a large high-risk PCa collective (cohort A) and its association to clinical data. A. Box- and whisker-plot of relative expression of *let-7a/b/c* in 6 BPH (white bar) and 98 high-risk PCa (grey bar) tissue specimens. Expression was assessed by qRT-PCR. B. Figure 3B summarizes the median expression of *let-7a*/*b*/*c* in 6 BPH and 98 PCa samples and shows corresponding standard deviation and the p value. The median expression of the BPH samples was used as threshold. The percentage of PCa samples with down-regulated *let-7* expression are shown in the 6th column of the table. C. Box- and whisker-plot show expression of *let-7b* and *-7c* in 98 high-risk PCa tissue and 6 BPH specimens; assessed by qRT-PCR. Subgroups are based on: • pathologic tumor features like Gleason Score and pathological tumor stage (GS ≤7, pT ≤3a – light grey), (GS ≥8, pT ≥3b – dark grey) and • clinic-pathological characteristics like BCR, CF, CRD (no BCR/no CF/no CRD – light grey), (BCR/CF/CRD – dark grey). A. + C. *Let-7a*/*b*/and *-c* expression is shown as means with error bars for standard deviation. *indicates p<0.01 (A) or p<0.05 (C). p values were calculated using the Welch 2 sample t-test.

We now wanted to confirm these results by qRT-PCR experiments on our entire high-risk cohort A (n = 98). Statistical analysis of the qRT-PCR data confirmed a significant down-regulation of *let-7a-c* in the high-risk PCa cases compared to benign hyperplasia (p≤0.001) (Fig. 3A). In about 81/84/and 96% of the tissue samples analyzed, the expression of *let-7a/b* and *-c,* respectively, was below the median expression of the BPH samples (Fig. 3B), indicating the tumor-specific value of the *let-7* family members in patients with high-risk prostate cancer.

### Down-regulation of *let-7b* is Associated with Aggressive Cancer Characteristics

Based on the assumption that some *let-7* family members are progressively down-regulated in the more aggressive PCa cases (group 2), we postulated that down-regulation of *let-7* might also be associated with clinic-pathological features or prognosis of PCa patients. Therefore, we looked for correlation between *let-7a/b/c* expression and tumor characteristics of the PCa collective A like Gleason Score (7≤ GS ≥8), pathological tumor stage (3a ≤pT ≥3b) and clinical endpoints like BCR, CF, and CRD (see Table 2). We found progressive down-regulation of *let-7b* in patients with poor cancer characteristics like high Gleason Score (≥8), biochemical relapse and clinical failure (p<0.05) (Fig. 3C). However, expression of *let-7c* was correlated with CF only. No correlation was seen between *let-7b* or *-7c* expression and pathological tumor stage (3a ≤pT ≥3b) or CRD (Fig. 3C). Since *let-7a* lacked any significant results in terms of cancer characteristics and clinical endpoints we neglected this miR in our further analyses (Fig. S2).

### *Let-7b* and *let-7c* as Prognostic Marker for Progression in a High-risk PCa Collective (cohort A)

**Figure 4:**
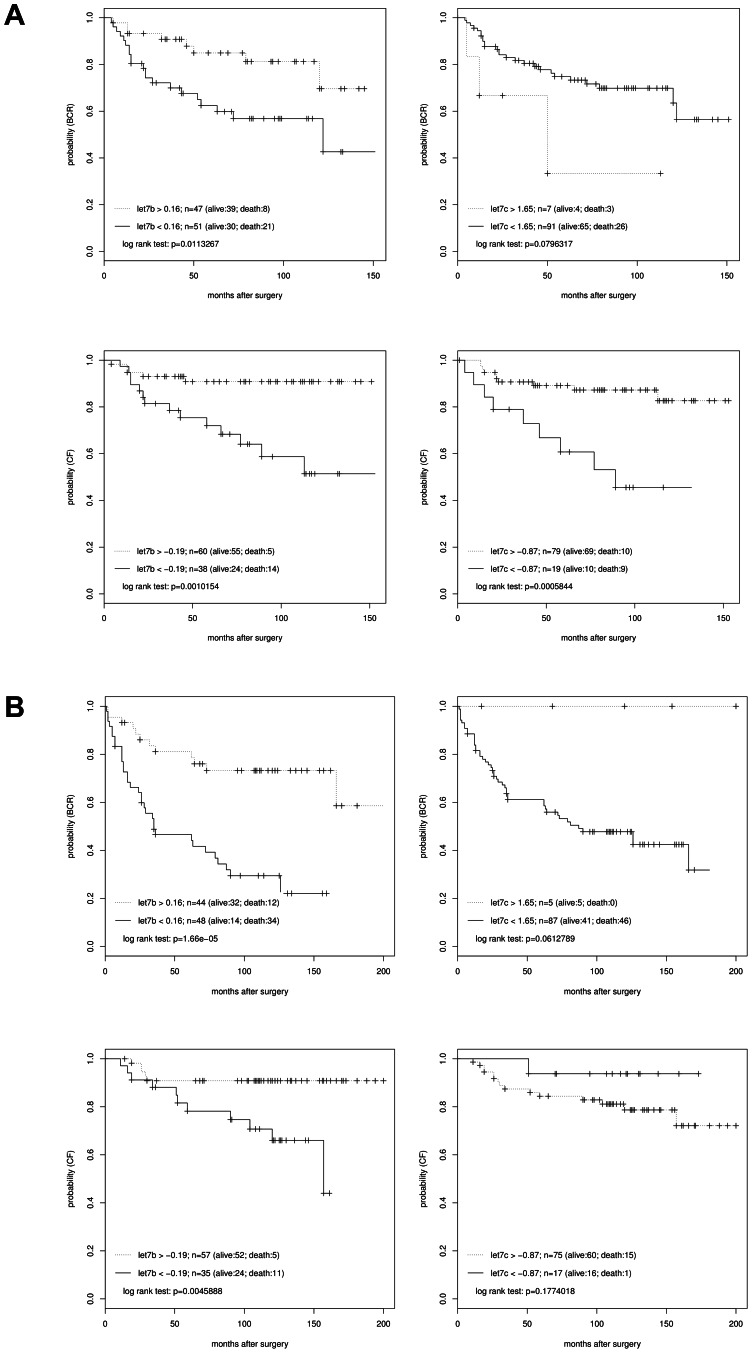
Kaplan-Meier analysis for BCR and CF in a learning and testing high-risk PCa cohort. Kaplan-Meier analysis for biochemical and clinical relapse of 98 and 92 patients with high-risk disease (cohort A and B). Groups were dichotomized in low and high *let-7b* and -*7c* expression. Survival curves were generated using the Bioconductor package “survival”. A. Kaplan Meier analysis of cohort A, the learning data set (n = 98). B. Kaplan Meier analysis of cohort B, the testing data set (n = 92). Low *let-7b* expression is associated with BCR and CF.

To determine whether *let-7b* or *-7c* could serve as a prognostic indicator for biochemical relapse or clinical failure we dichotomized a high-risk study group (cohort A) in low and high *let-7b/c* expression based on z-scores. Kaplan-Meier estimates showed a significant difference between groups of high and low *let-7b* expression in BCR (log rank p = 0.01), while *let-7c* showed borderline significance (log rank p = 0.08) (Fig. 4A). The 10 yr biochemical progression-free survival rate was 65% and 37% for high and low *let-7b* expression, respectively. For *let-7b* and *let-7c* Kaplan-Meier estimates also predicted a significant difference between groups of high and low expression in CF (log rank p<0.001) (Fig. 4A). 14 of 38 patients (37%) with low *let-7b* expression, but only 5 of 60 patients (8.3%) in the high *let-7b* expression group were found to have experienced CF.

Cox regression analysis showed that *let-7b* but not *let-7c* expression was univariately significant for the prediction of BCR (HR 0.36 (0.16–0.82)). When *let-7b* expression and Gleason Score were considered together in a multivariate model both variables independently predicted BCR. Eventually, we evaluated whether *let-7b* or *let-7c* are independent markers for clinical failure in cohort A. The expression of both miRs are univariately significant for the prediction of clinical failure (Table 3). However, in a multivariate Cox regression model only high Gleason Score but not *let-7b* or -*c* were significantly correlated to poor prognosis.

**Table 3:** Uni- and multivariate Cox regression analysis of high-risk cohort A.

variable + BCR		univariate			multivariate		
	n	HR	95% CI	p value	HR	95% CI	p value
*let-7b* dichot.	high 47, low 51	0.36	0.161 - 0.823	0.02	0.44	0.193 - 1.022	0.05
*let-7c* dichot.	high 7, low 91	2.81	0.840 - 9.388	0.09			
Gleason Score	98	1.62	1.149 - 2.296	< 0.01	1.50	1.059 - 2.125	0.02
comb. pT	98	1.58	0.717 - 3.496	0.26			
pre.PSA	98	1.01	0.999 - 1.021	0.07			
**variable + CF**		**univariate**			**multivariate**		
	**n**	**HR**	**95% CI**	**p value**	**HR**	**95% CI**	**p value**
*let-7b* dichot.	high 47, low 51	0.21	0.076 - 0.588	< 0.01	0.46	0.148 - 1.411	0.17
*let-7c* dichot.	high 7, low 91	0.23	0.095 - 0.578	< 0.01	0.53	0.189 - 1.475	0.22
Gleason Score	98	2.93	1.741 - 4.916	< 0.01	2.15	1.276 - 3.615	< 0.01
comb. pT	98	2.05	0.737 - 5.683	0.17			
pre.PSA	98	1.02	1.003 - 1.028	0.01	1.01	0.997 - 1.024	0.13

In summary the Kaplan Meier estimates and Cox regression analysis indicate that low *let-7b* and *let-7c* expression might be correlated to BCR and CF in cohort A.

### Validation of *let-7b* and *let-7c* as Prognostic Markers in an Independent, External Testing Collective (cohort B)

To validate the role of *let-7b* and -*c* as prognostic markers we worked with an independent collective of primary high-risk PCa cases (cohort B). As already observed for cohort A we detected a significant down-regulation of *let-7b* and *let-7c* in PCa samples when compared to BPHs (Table S2). We created z-score levels based on the analyzed ΔC_t_ expression data of both *let-7*s in the testing cohort. Subsequently we dichotomized the testing cohort using the cut-off levels identified for collective A. Each sample was predicted to be either on high or low risk for BCR or CF based on these thresholds. We could not confirm the prognostic role of *let-7c* as marker for progression in the testing cohort as indicated by Kaplan Meier estimates and Cox regression analysis (Fig. 4b and Table 4). Nevertheless, Kaplan Meier estimates indicated that patients with low *let-7b* expression showed significantly shorter cumulative time to BCR or CF than those with high *let-7b* expression (Fig. 4b). To identify whether *let-7b* is an independent prognostic covariate for progression of PCa we performed Cox proportional hazard analysis. The multivariate Cox regression model revealed *let-7b*, but not *let-7c*, to be an independent prognostic marker for both: BCR (HR = 0.3 (0.15–0.61)) and CF (HR = 0.23 (0.08–0.70) (Table 4).

**Table 4:** Uni- and multivariate Cox regression analysis of high-risk cohort B

variable + BCR		univariate			multivariate		
	n	HR	95% CI	p value	HR	95% CI	p value
*let-7b* dichot.	high 44, low 48	0.25	0.125 - 0.493	< 0.01	0.30	0.152 - 0.610	< 0.01
*let-7c* dichot.	high 5, low 87	0.00	0 - Inf	1.00			
Gleason Score	92	1.54	1.169 - 2.022	< 0.01	1.36	1.012 - 1.820	0.04
comb. pT	92	2.20	1.221 - 3.968	< 0.01	1.24	0.640 - 2.398	0.53
pre.PSA	92	1.02	1.002 - 1.030	0.03	1.01	0.995 - 1.024	0.19
**variable + CF**		**univariate**			**multivariate**		
	**n**	**HR**	**95% CI**	**p value**	**HR**	**95% CI**	**p value**
*let-7b* dichot.	high 44, low 48	0.24	0.084 - 0.703	< 0.01	0.23	0.078 - 0.700	< 0.01
*let-7c* dichot.	high 5, low 87	3.67	0.485 - 27.80	0.21			
Gleason Score	92	3.98	2.327 - 6.823	< 0.01	4.00	2.076 - 7.712	< 0.01
comb. pT	92	5.38	1.946 - 14.84	< 0.01	0.88	0.801 - 7.220	0.12
pre.PSA	92	1.02	1.00 - 1.041	0.05			

### *HMGA1* is a Direct Target of *let-7b* in PCa Cells

**Figure 5:**
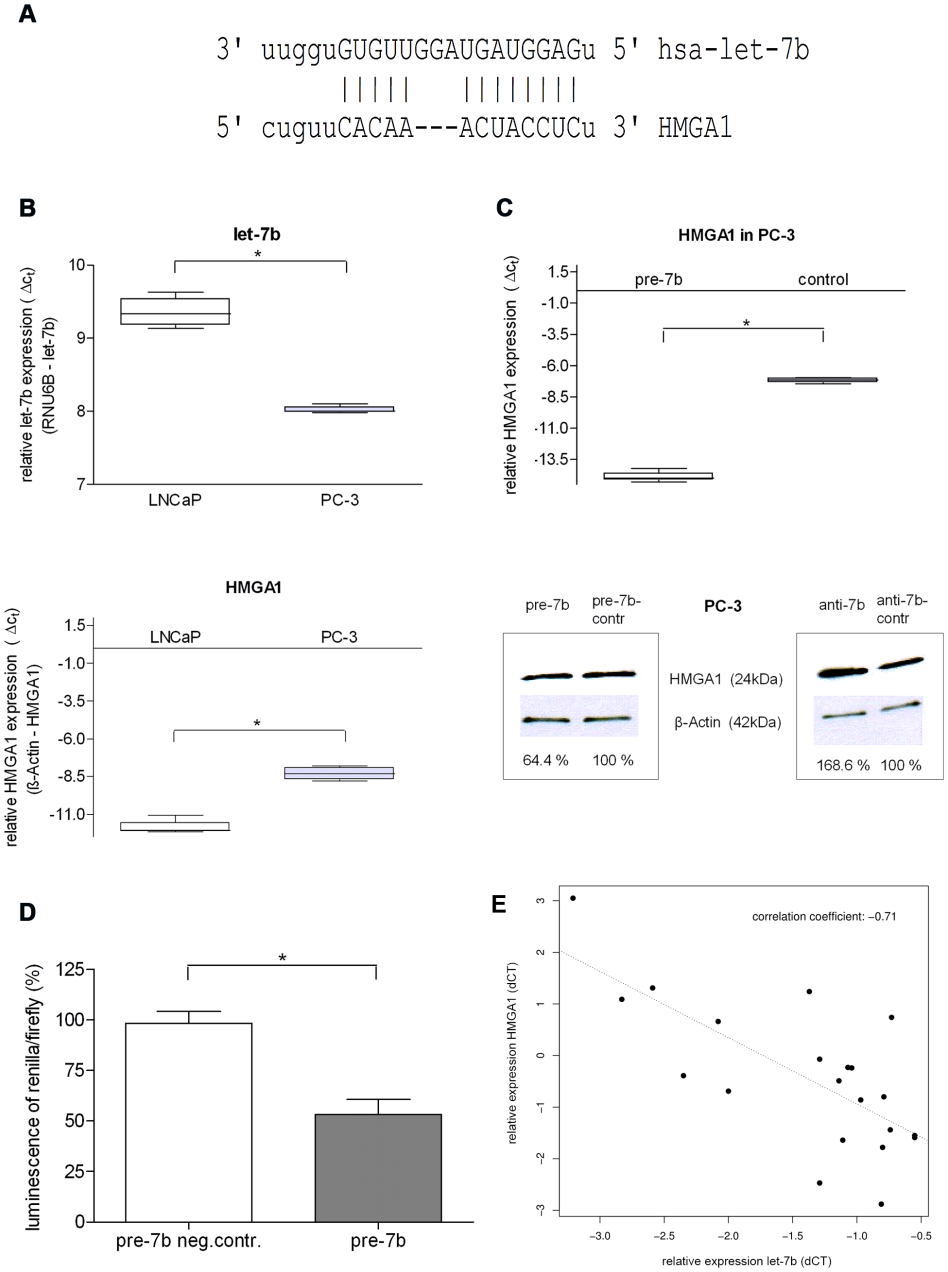
*HMGA1* is a direct target of *let-7b* in prostate cancer. A. 3′UTR-sequence of binding sites for *let-7b* are indicated by vertical lines towards *HMGA1*. B. qRT-PCR for *let-7b* and *HMGA1* in untreated LNCaP and PC-3 cells. While *let-7b* shows higher expression in LNCaP compared to PC-3 cells, *HMGA1* expression shows inverse expression to *let-7b*. C. qRT-PCR and Western Blot analysis for *HMGA1* using lysates from PC-3 cells transiently transfected with pre−/anti-*7b* and negative control, respectively. Western Blot analysis was performed in two independent experiments. Band intensity was measured by Image J in regard to housekeeping gene (*β-Actin*) band intensity. D. *HMGA1* as target of *let-7b*. Luciferase construct containing the 3′UTR of *HMGA1* were transfected with either pre–*let-7b* or pre-miR control into PC-3 cells. Relative expression of firefly luciferase was determined 48 h post transfection and normalized to the transfection control. Error bars represent +/− standard deviation and were calculated using three independent experiments. E. Correlation analysis of *HMGA1* and *let-7b* in vivo. In 21 pairs of PCa and adjacent benign tissue qRT-PCR analysis for *let-7b* and *HMGA1* were performed. Expression levels, normalized by *RNU6B* or *β-Actin*, were used to calculate the Pearson’s correlation coefficient (r = -0.71). B. + C. Relative expression is shown by means of 3 independent qRT-PCR experiments. *indicate statistically significant results (p<0.05). P values were calculated using the Welch 2 sample t-test.

Expression and correlation studies of the high-risk cohort had revealed *let-7b* to be a potential prognostic marker for progression in high-risk PCa. Therefore, we searched for potential new target genes using database target search programs (pictar, miRanda). A binding site for *let-7b* was found in the 3′UTR of *HMGA1* suggesting that this oncogene might be a target of *let-7b* (Fig. 5A). To determine the relative level of *let-7b* and *HMGA1* in prostate cancer cell lines we performed qRT-PCR on LNCaP and PC-3 cells. We confirmed results from a previous study showing that *let-7b* is expressed at very low levels in PC-3 when compared to hormone sensitive LNCaP cells [18]. Our results showed that *HMGA1* expression is reduced in LNCaP when compared to PC-3 cells indicating a reciprocal expression of *HMGA1* and *let-7b* in these PCa cell lines (Fig. 5B).

In addition, we analyzed the *HMGA1* expression in PC-3 cells 48 hours after transient transfection with pre- and anti-*let-7b* by qRT-PCR and Western Blot analysis. *HMGA1*-mRNA expression was significantly lower after *let-7b* over-expression (p = 0.004) (Fig. 5C). On protein level an inverse correlation was detected by Western Blot analysis in PC-3 cells. Reintroduction of *let-7b* lead to diminished *HMGA1* protein expression compared to cells transfected with pre-negative control (down-regulation to 64.4% in PC-3). On the other hand, suppression of *let-7b* in these cancer cells resulted in an up-regulation of *HMGA1* up to 168.6% (Fig. 5C).

We performed luciferase reporter assay in LNCaP cells and found the relative luciferase activity to be markedly diminished after *let-7b* co-transfection. These results indicate that *let-7b* interferes with *HMGA1* translation via direct interaction with most downstream located *let-7b* complementary sites of the 3′UTR (Fig. 5D). We herewith confirm *HMGA1* to be a direct target of *let-7b*.

To compare whether *HMGA1* expression was correlated to *let-7b* in primary human PCa we isolated m(i)RNA of PCa and adjacent benign tissue samples (cohort C) and performed qRT-PCR analyses on *let-7b* and *HMGA1*. We utilized 21 pairs of PCa samples in which expression of *let-7b* was significantly suppressed (≥0.5 cycles) in cancerous compared to benign tissue (Fig. S3). The balance of m(i)RNA expression in PCa and BPH was used for assessment of the correlation coefficient (ΔC_t_ PCa*_let-7b/HMGA1_* - ΔC_t_ benign tissue *_let-7b/HMGA1_*). Pearson’s correlation coefficient confirmed a negative correlation between *HMGA1* and *let-7b* expression (r = −0.71) suggesting that expression of *HMGA1* in primary PCa cases is at least partially regulated by *let-7b* (Fig. 5E).

## Discussion

Over the last years specific miRNA signatures for PCa have been described in several studies suggesting that miRNAs or miRNA profiles can be used as diagnostic or prognostic markers in this disease [5]–[9], [11], [12], [15], [16]. Nevertheless, there is not much knowledge about the applicability of these as prognostic markers in PCa. To screen for miRNAs that show specific expression for high-risk disease we analyzed 13 high-risk PCa samples by a microarray experiment. Initially, we compared the expression of all 13 high-risk samples to the expression of 6 BPH samples and found a miRNA expression profile that allowed a clear distinction between the malignant and the benign disease. This scatter, with the majority of miRNAs down-regulated in the high-risk PCa samples, was comparable to results seen in further microarray analyses using contemporary PCa study groups. Among the most highly de-regulated microRNAs we primarily found miRNAs that were previously described as PCa specific in other studies e.g. *miR-16, -26a, -125b*, *-143*, *-205*, and *miR-221*, indicating that the high-risk PCa cases show comparable miRNA expression alterations as observed in contemporary PCa collectives [6]–[12].

Subsequently, we analyzed the miRNA expression alterations among PCa samples with and without CF to screen for prognostic relevant miRNAs. Interestingly, hierarchical clustering of microarray data using a selected set of miRs differentially expressed in BPH and PCa samples generated a tree with additional separation of these both PCa groups suggesting that high-risk PCa cases with early CF could be characterized by a distinct miRNA expression profile. As expected from these results, we found seven miRNAs that were differentially expressed between both PCa groups and the non-malignant BPH samples. Three of these, namely *miR-361*, *-515-3* and -*515-5,* have not been described as oncomiRs until now and their relevance in development and progression of PCa remains to be tested in the future. Four other miRNAs (*miR-146b*, *-181b*, *let-7a* and *let-7c*) are known oncogenic or tumor suppressor miRNAs [30]–[33]. Our results demonstrate that *miR-146b* and *-181b* are down-regulated in all PCa cases, but showed up-regulation when we compared expression in PCa patients with CF to patients with CPFS. These results are supported by studies implicating that *miR-181b* up-regulation has prognostic effects in cancer patients: Expression of *miR-181* is significantly lower in low-risk myelodysplastic syndrome (MDS), whereas patients with high-risk MDS show an elevated expression of this miRNA [30]. It was also demonstrated that squamous cell lung cancer patients with high *miR-146b* expression have significantly worse overall survival compared to patients with low expression [31]. Moreover, down-regulation of *miR-146b* is described to correlate with development of hormone refractory PCa [32]. Altogether, these results implicate that deregulation of *miR-181b* or *miR-146b* might be critically involved in development and progression of PCa.

However, our microarray-based miRNA expression study is limited to a low number of analyzed cases, since it was primarily designed as a screening approach. To test the clinical relevance of the identified candidate miRNAs as prognostic markers larger cohorts have to be tested. In addition our expression profiling is limited by the use of BPH samples as control tissue. Based on the highly invasive growth of various high-risk PCa cases (data not shown) we were not able to isolate adjacent normal prostate tissue with sufficient amounts of benign prostatic ducts for analyses. We are aware that gene expression in BPH and cancer samples might differ in regard to the different origin of the tissues (transition zone vs. peripheral zone). Therefore, the results obtained by the microarray study need to be interpreted cautiously and await further experimental clarification by validation in independent study cohorts and by functional studies in the future. However, our current study focused on expression differences of *let-7* family members between samples of cancer patients with diverse outcome rather than between cancerous and non-cancerous tissue.

Thus, we analyzed the expression of *let-7* family members in a large high-risk PCa collective to elucidate their potential role as prognostic biomarker. As expected from the microarray data we saw down-regulation of *let-7a/b* and *-c* in the majority of the high-risk PCa cases analyzed. However, *let-7a* showed no clear impact as prognostic marker in association analysis with clinical parameters. Even though, we found some correlation of low *let-7c* expression with progression of PCa in a high-risk cohort, we could not validate its role as prognostic marker for biochemical or clinical progression using an independent testing cohort. We therefore concluded that *let-7c* is frequently down-regulated in PCa, but its clinical use as prognostic marker is very limited. With regard to the role of *let-7b* as potential prognostic marker we showed that patients with poor cancer characteristics like BCR or CF have lower *let-7b* expression than patients with better clinical outcome. As prognostic factor *let-7b* is independent from other tested clinical factors and is closely related to PSA relapse and recurrence of the disease. Altogether our findings suggest that reduced expression of *let-7-a/b* and *-c* may play a role in pathogenesis of prostate cancer and strongly support that reduced *let-7b* expression may have a prognostic impact on biochemical and clinical progression of PCa patients.

Our results implicating that *let-7b* is a useful biomarker to predict clinical outcome are in line with studies in patients with lung cancer. Takamizawa et al. analyzed tissue specimen of 143 lung cancer patients and found that patients, irrespective of disease stage, had significantly worse post-operative survival, if *let-7* was down-regulated [19]. In addition, the potential of *let-7* as biomarker in various cancer types was recently implicated in a systematic review demonstrating that *let-7* is associated most frequently and significantly with clinical outcome parameters in cancer patients [34]. Martens-Uzunova and colleagues recently also found *let-7b* to be significantly dys-regulated in PCa. They created a miRNA-derived prognostic predictor, consisting of 25 miRNAs, including *let-7b*, that forecasted post-operative outcome [10]. There is also clear evidence that members of the *let-7* family function as tumor suppressor in various cancer types and that over-expression of *let-7* suppresses growth of cancer cells in vivo and in vitro including PCa [33]. More recently down-regulation of *let-7* was also demonstrated in prostate cancer stem cells indicating an important role of this miRNA in tumorigenicity [25]. Altogether, this further supports our findings of *let-7b* being an important predictor in clinical outcome in various cancer types including PCa.

Down-regulation of several *let-*7 isoforms in high-risk disease indicates their potential involvement in pathogenesis and progression of PCa. But how *let-7* family members contribute to this is poorly understood. It was previously shown that members of this family simultaneously inhibit multiple oncogenic pathways in cancer cells regulating the expression of relevant oncogenes like *cmyc*, *ras*, *CDC25A*, and *HMGA2* among others [22]–[24]. Moreover, it was shown that the developmentally regulated RNA-binding protein *Lin28* selectively blocks the maturation of *let-7* members, which in turn repress the expression of *Lin28* [35]. Based on the connection of *Lin28/let-7* several line of evidence support a role of *let-7* in stem cell biology and development. Deregulation of this feedback mechanism is critical in controlling self-renewal, a feature of cancer stem cells, which again is associated with aggressiveness and progression in various cancer types [36], [37]. Interestingly, it was shown that the developmentally regulated gene *HMGA1* is inversely correlated to *let-7* in gastro-pancreatic carcinoma and retinoblastoma [38], [39]. *HMGA1* is a member of the non-histone nuclear binding proteins that alter chromatin structure by inducing DNA confirmation and thus regulating the transcriptional activity of several genes [40]. *HMGA1* over-expression plays a critical role in carcinogenesis and its up-regulation was observed in many cancer entities like retinoblastoma, pancreatic, colorectal, lung and prostate cancer for example [41]–[46]. Increased levels of *HMGA1* were shown to be correlated with the development of high-grade tumors in various cancer types [47] and more recently it was demonstrated that overexpression of *HMGA1* is also associated with late stage prostate cancer [48]. Furthermore, over-expression of *HMGA1* in PCa cells was associated with enhanced proliferation and metastasis as well as with development of androgen-independent growth [49]. Interestingly, it was recently shown that *HMGA1* is regulated by *miR-296*, but not by *let-7c* [48]. We report in the current study that *let-7b* represses *HMGA1* expression by inhibiting translation and additionally showed that *HMGA1* is a direct target of *let-7b*. We performed correlation studies ex vivo on primary PCa specimens and confirmed an inverse relation between *let-7b* and its target *HMGA1*. Based on these data we suggest that *let-7b* mediated regulation of *HMGA1* might partially explain the role of *let-7b* in progression of PCa in context with its role in the development of cancer stem cells. Identification of the interaction between *let-7b* and *HMGA1* will provide promising therapeutic opportunities in PCa treatment.

In summary, we defined a distinct miRNA expression profile in high-risk PCa with early CF. Our study further gives evidence that one of the detected miRNAs, namely *let-7b,* works as a relevant prognostic biomarker predicting clinical recurrence in high-risk PCa. We identified the oncogene *HMGA1* as direct target of *let-7b* in PCa and found an inverse correlation of both in primary PCa cases. It will be of great interest to understand the role of *let-7b/HMGA1* interaction for the development of cancer stem cells in context with the regulation of others potential target genes of the *let-7* family members. Altogether, our study represents a basis to develop novel strategies for the prognosis and therapy of PCa patients at high risk for clinical recurrence of the disease.

## Supporting Information

Figure S1 **MiRNA expression in high-risk PCa (cohort A) with early clinical failure.** Venn diagram showing relationships between human miRs that were expressed significantly different (±1.5 fold) in PCa group 1 vs. PCa group 2 vs. BPH (adj p<0.05). Circles include numbers of up- or down-regulated human miRs of each pair-wise comparison. Common miRs between different comparisons are shown in intersections. The table below lists all human miRs in which expression was significantly different either: between (gr.1 vs. gr.2) (blue), (gr.2 vs. BPH) (red), (gr.1 vs. BPH) (green), between (gr. 2 vs. BPH) and (gr. 1 vs. BPH) (yellow). (Complementary data to Fig. 2.) All miRNAs are ranked based on the maximum expression fold change.
(XLSX)

Figure S2 **Association of** ***let-7a*** **expression and clinical data.** Box- and whisker-plot show expression of *let-7a* in 98 high-risk PCa tissue and 6 BPH specimens; assessed by qRT-PCR. Subgroups are based on: • pathologic tumor features like Gleason Score and pathological tumor stage (GS ≤7, pT ≤3a–light grey), (GS ≥8, pT ≥3b–dark grey) and • clinic-pathological characteristics like BCR, CF, CRD (no BCR/no CF/no CRD–light grey), (BCR/CF/CRD–dark grey). *Let-7a* expression is not associated to clinical data in high-risk PCa. Expression is shown as means with error bars for standard deviation. p values were calculated using the Welch 2 sample t-test.
(TIFF)

Figure S3 ***Let-7b*** **expression in cancerous and adjacent benign prostatic tissue (cohort C).** Scatter Plot shows expression of *let-7b* in 21 pairs of PCa tissue and adjacent benign tissue. Median expression of *let-7b* is significantly lower in the cancerous compared to the benign tissue. p values were calculated using the Welch 2 sample t-test.
(TIFF)

Table S1 **MiRNA expression in high-risk PCa (cohort A) with early clinical failure compared to expression in BPH.** Microarray analysis of 6 BPH and 13 high-risk PCa tissue specimens (cohort A). The table lists all 82 differentially expressed miRNAs (fold change >1.5 and an adjusted p value <0.05) The majority of miRNAs showed decreased expression in high-risk PCa compared to benign prostatic tissue.
(XLSX)

Table S2 **MiRNA expression in high-risk PCa (cohort B) and BPH.** Table 2 summarizes the median expression of *let-7b*/*c* in 6 BPH and 92 high-risk PCa samples and shows corresponding standard deviation and the p value. The median expression of the BPH samples was used as threshold. The percentage of PCa samples with down-regulated *let-7* expression are shown in the 6th column of the table.
(XLSX)

## References

[1] Ferlay J, Autier P, Boniol M, Heanue M, Colombet M, et al (2007) Estimates of the cancer incidence and mortality in Europe in 2006. Ann Oncol 18: 581–592. 17287242 10.1093/annonc/mdl498

[2] Petrovich Z, Lieskovsky G, Stein JP, Huberman M, Skinner DG (2002) Comparison of surgery alone with surgery and adjuvant radiotherapy for pT3N0 prostate cancer. BJU Int 89: 604–611. 11942974 10.1046/j.1464-410x.2002.02698.x

[3] Spahn M, Joniau S, Gontero P, Fieuws S, Marchioro G, et al.. (2010) Outcome predictors of radical prostatectomy in patients with prostate-specific antigen greater than 20 ng/ml: a European multi-institutional study of 712 patients. Eur Urol 58: 1–7; discussion 10–11. 10.1016/j.eururo.2010.03.001 20299147

[4] Wilt TJ, Brawer MK, Jones KM, Barry MJ, Aronson WJ, et al (2012) Radical prostatectomy versus observation for localized prostate cancer. N Engl J Med 367: 203–213. 22808955 10.1056/NEJMoa1113162 PMC3429335

[5] Calin GA, Croce CM (2006) MicroRNA signatures in human cancers. Nat Rev Cancer 6: 857–866. 17060945 10.1038/nrc1997

[6] Porkka KP, Pfeiffer MJ, Waltering KK, Vessella RL, Tammela TL, et al (2007) MicroRNA expression profiling in prostate cancer. Cancer Res 67: 6130–6135. 17616669 10.1158/0008-5472.CAN-07-0533

[7] Catto JW, Alcaraz A, Bjartell AS, De Vere White R, Evans CP, et al (2011) MicroRNA in prostate, bladder, and kidney cancer: a systematic review. Eur Urol 59: 671–681. 21296484 10.1016/j.eururo.2011.01.044

[8] Lu J, Getz G, Miska EA, Alvarez-Saavedra E, Lamb J, et al (2005) MicroRNA expression profiles classify human cancers. Nature 435: 834–838. 15944708 10.1038/nature03702

[9] Ozen M, Creighton CJ, Ozdemir M, Ittmann M (2008) Widespread deregulation of microRNA expression in human prostate cancer. Oncogene 27: 1788–1793. 17891175 10.1038/sj.onc.1210809

[10] Martens-Uzunova ES, Jalava SE, Dits NF, van Leenders GJ, Moller S, et al (2012) Diagnostic and prognostic signatures from the small non-coding RNA transcriptome in prostate cancer. Oncogene 31: 978–991. 21765474 10.1038/onc.2011.304

[11] Wach S, Nolte E, Szczyrba J, Stohr R, Hartmann A, et al (2012) MicroRNA profiles of prostate carcinoma detected by multiplatform microRNA screening. Int J Cancer 130: 611–621. 21400514 10.1002/ijc.26064

[12] Schaefer A, Jung M, Mollenkopf HJ, Wagner I, Stephan C, et al (2010) Diagnostic and prognostic implications of microRNA profiling in prostate carcinoma. Int J Cancer 126: 1166–1176. 19676045 10.1002/ijc.24827

[13] Saini S, Majid S, Yamamura S, Tabatabai L, Suh SO, et al (2011) Regulatory Role of mir-203 in Prostate Cancer Progression and Metastasis. Clin Cancer Res 17: 5287–5298. 21159887 10.1158/1078-0432.CCR-10-2619

[14] Hulf T, Sibbritt T, Wiklund ED, Patterson K, Song JZ, et al.. (2012) Epigenetic-induced repression of microRNA-205 is associated with MED1 activation and a poorer prognosis in localized prostate cancer. Oncogene. 10.1038/onc.2012.300 22869146

[15] Larne O, Martens-Uzunova E, Hagman Z, Edsjo A, Lippolis G, et al.. (2012) miQ-A novel microRNA based diagnostic and prognostic tool for prostate cancer. Int J Cancer. 10.1002/ijc.27973 23184647

[16] Spahn M, Kneitz S, Scholz CJ, Stenger N, Rudiger T, et al (2010) Expression of microRNA-221 is progressively reduced in aggressive prostate cancer and metastasis and predicts clinical recurrence. Int J Cancer 127: 394–403. 19585579 10.1002/ijc.24715

[17] Kong D, Heath E, Chen W, Cher ML, Powell I, et al (2012) Loss of Let-7 Up-Regulates EZH2 in Prostate Cancer Consistent with the Acquisition of Cancer Stem Cell Signatures That Are Attenuated by BR-DIM. PLoS One 7: e33729. 22442719 10.1371/journal.pone.0033729 PMC3307758

[18] Nadiminty N, Tummala R, Lou W, Zhu Y, Shi XB, et al (2012) MicroRNA let-7c Is Downregulated in Prostate Cancer and Suppresses Prostate Cancer Growth. PLoS One 7: e32832. 22479342 10.1371/journal.pone.0032832 PMC3316551

[19] Takamizawa J, Konishi H, Yanagisawa K, Tomida S, Osada H, et al (2004) Reduced expression of the let-7 microRNAs in human lung cancers in association with shortened postoperative survival. Cancer Res 64: 3753–3756. 15172979 10.1158/0008-5472.CAN-04-0637

[20] Dahiya N, Sherman-Baust CA, Wang TL, Davidson B, Shih Ie M, et al (2008) MicroRNA expression and identification of putative miRNA targets in ovarian cancer. PLoS One 3: e2436. 18560586 10.1371/journal.pone.0002436 PMC2410296

[21] Qian PX, Zuo ZH, Wu ZS, Meng XY, Li GP, et al (2011) Pivotal Role of Reduced let-7g Expression in Breast Cancer Invasion and Metastasis. Cancer Res 71: 6463–6474. 21868760 10.1158/0008-5472.CAN-11-1322

[22] Johnson SM, Grosshans H, Shingara J, Byrom M, Jarvis R, et al (2005) RAS is regulated by the let-7 microRNA family. Cell 120: 635–647. 15766527 10.1016/j.cell.2005.01.014

[23] Mayr C, Hemann MT, Bartel DP (2007) Disrupting the pairing between let-7 and Hmga2 enhances oncogenic transformation. Science 315: 1576–1579. 17322030 10.1126/science.1137999 PMC2556962

[24] Kim HH, Kuwano Y, Srikantan S, Lee EK, Martindale JL, et al (2009) HuR recruits let-7/RISC to repress c-Myc expression. Genes Dev 23: 1743–1748. 19574298 10.1101/gad.1812509 PMC2720259

[25] Liu C, Kelnar K, Vlassov AV, Brown D, Wang J, et al (2012) Distinct microRNA expression profiles in prostate cancer stem/progenitor cells and tumor-suppressive functions of let-7. Cancer Res 72: 3393–3404. 22719071 10.1158/0008-5472.CAN-11-3864 PMC3872033

[26] Nadiminty N, Tummala R, Lou W, Zhu Y, Zhang J, et al (2012) MicroRNA let-7c suppresses androgen receptor expression and activity via regulation of Myc expression in prostate cancer cells. J Biol Chem 287: 1527–1537. 22128178 10.1074/jbc.M111.278705 PMC3256915

[27] Garg M (2012) MicroRNAs, stem cells and cancer stem cells. World J Stem Cells 4: 62–70. 22993663 10.4252/wjsc.v4.i7.62 PMC3443713

[28] Huber W, von Heydebreck A, Sultmann H, Poustka A, Vingron M (2002) Variance stabilization applied to microarray data calibration and to the quantification of differential expression. Bioinformatics 18 Suppl 1S96–104. 12169536 10.1093/bioinformatics/18.suppl_1.s96

[29] Gentleman RC, Carey VJ, Bates DM, Bolstad B, Dettling M, et al (2004) Bioconductor: open software development for computational biology and bioinformatics. Genome Biol 5: R80. 15461798 10.1186/gb-2004-5-10-r80 PMC545600

[30] Sokol L, Caceres G, Volinia S, Alder H, Nuovo GJ, et al (2011) Identification of a risk dependent microRNA expression signature in myelodysplastic syndromes. Br J Haematol 153: 24–32. 21332710 10.1111/j.1365-2141.2011.08581.x PMC4294220

[31] Raponi M, Dossey L, Jatkoe T, Wu X, Chen G, et al (2009) MicroRNA classifiers for predicting prognosis of squamous cell lung cancer. Cancer Res 69: 5776–5783. 19584273 10.1158/0008-5472.CAN-09-0587

[32] Man YG, Fu SW, Liu AJ, Stojadinovic A, Izadjoo MJ, et al (2011) Aberrant expression of chromogranin A, miR-146a, and miR-146b-5p in prostate structures with focally disrupted basal cell layers: an early sign of invasion and hormone-refractory cancer? Cancer Genomics Proteomics 8: 235–244. 21980038

[33] Johnson CD, Esquela-Kerscher A, Stefani G, Byrom M, Kelnar K, et al (2007) The let-7 microRNA represses cell proliferation pathways in human cells. Cancer Res 67: 7713–7722. 17699775 10.1158/0008-5472.CAN-07-1083

[34] Nair VS, Maeda LS, Ioannidis JP (2012) Clinical outcome prediction by microRNAs in human cancer: a systematic review. J Natl Cancer Inst 104: 528–540. 22395642 10.1093/jnci/djs027 PMC3317879

[35] Viswanathan SR, Daley GQ, Gregory RI (2008) Selective blockade of microRNA processing by Lin28. Science 320: 97–100. 18292307 10.1126/science.1154040 PMC3368499

[36] Yang X, Lin X, Zhong X, Kaur S, Li N, et al (2010) Double-negative feedback loop between reprogramming factor LIN28 and microRNA let-7 regulates aldehyde dehydrogenase 1-positive cancer stem cells. Cancer Res 70: 9463–9472. 21045151 10.1158/0008-5472.CAN-10-2388 PMC3057570

[37] Ji J, Wang XW (2010) A Yin-Yang balancing act of the lin28/let-7 link in tumorigenesis. J Hepatol 53: 974–975. 20739081 10.1016/j.jhep.2010.07.001 PMC2949515

[38] Rahman MM, Qian ZR, Wang EL, Sultana R, Kudo E, et al (2009) Frequent overexpression of HMGA1 and 2 in gastroenteropancreatic neuroendocrine tumours and its relationship to let-7 downregulation. Br J Cancer 100: 501–510. 19156147 10.1038/sj.bjc.6604883 PMC2658538

[39] Mu GY, Liu H, Zhou F, Xu XY, Jiang H, et al (2010) Correlation of overexpression of HMGA1 and HMGA2 with poor tumor differentiation, invasion, and proliferation associated with let-7 down-regulation in retinoblastomas. Hum Pathol 41: 493–502. 20004941 10.1016/j.humpath.2009.08.022

[40] Bianchi ME, Agresti A (2005) HMG proteins: dynamic players in gene regulation and differentiation. Curr Opin Genet Dev 15: 496–506. 16102963 10.1016/j.gde.2005.08.007

[41] Fusco A, Fedele M (2007) Roles of HMGA proteins in cancer. Nat Rev Cancer 7: 899–910. 18004397 10.1038/nrc2271

[42] Mu G, Liu H, Zhou F, Xu X, Jiang H, et al (2010) Correlation of overexpression of HMGA1 and HMGA2 with poor tumor differentiation, invasion, and proliferation associated with let-7 down-regulation in retinoblastomas. Hum Pathol 41: 493–502. 20004941 10.1016/j.humpath.2009.08.022

[43] Liau SS, Jazag A, Ito K, Whang EE (2007) Overexpression of HMGA1 promotes anoikis resistance and constitutive Akt activation in pancreatic adenocarcinoma cells. Br J Cancer 96: 993–1000. 17342093 10.1038/sj.bjc.6603654 PMC2360112

[44] Fedele M, Bandiera A, Chiappetta G, Battista S, Viglietto G, et al (1996) Human colorectal carcinomas express high levels of high mobility group HMGI(Y) proteins. Cancer Res 56: 1896–1901. 8620511

[45] Sarhadi VK, Wikman H, Salmenkivi K, Kuosma E, Sioris T, et al (2006) Increased expression of high mobility group A proteins in lung cancer. J Pathol 209: 206–212. 16521118 10.1002/path.1960

[46] Tamimi Y, van der Poel HG, Denyn MM, Umbas R, Karthaus HF, et al (1993) Increased expression of high mobility group protein I(Y) in high grade prostatic cancer determined by in situ hybridization. Cancer Res 53: 5512–5516. 8221692

[47] Tamimi Y, van der Poel HG, Karthaus HF, Debruyne FM, Schalken JA (1996) A retrospective study of high mobility group protein I(Y) as progression marker for prostate cancer determined by in situ hybridization. Br J Cancer 74: 573–578. 8761372 10.1038/bjc.1996.403 PMC2074681

[48] Wei JJ, Wu X, Peng Y, Shi G, Basturk O, et al (2011) Regulation of HMGA1 expression by microRNA-296 affects prostate cancer growth and invasion. Clin Cancer Res 17: 1297–1305. 21138859 10.1158/1078-0432.CCR-10-0993 PMC3196617

[49] Takeuchi I, Takaha N, Nakamura T, Hongo F, Mikami K, et al (2012) High mobility group protein AT-hook 1 (HMGA1) is associated with the development of androgen independence in prostate cancer cells. Prostate 72: 1124–1132. 22213442 10.1002/pros.22460

