# Supplementary figures and images for: Distinct microRNA Expression Profile in Prostate Cancer Patients with Early Clinical Failure and the Impact of let-7 as Prognostic Marker in High-Risk Prostate Cancer
Source: PLoS One. 2013 Jun 14;8(6):e65064. doi: 10.1371/journal.pone.0065064 (PMC3683014; doi:10.1371/journal.pone.0065064)

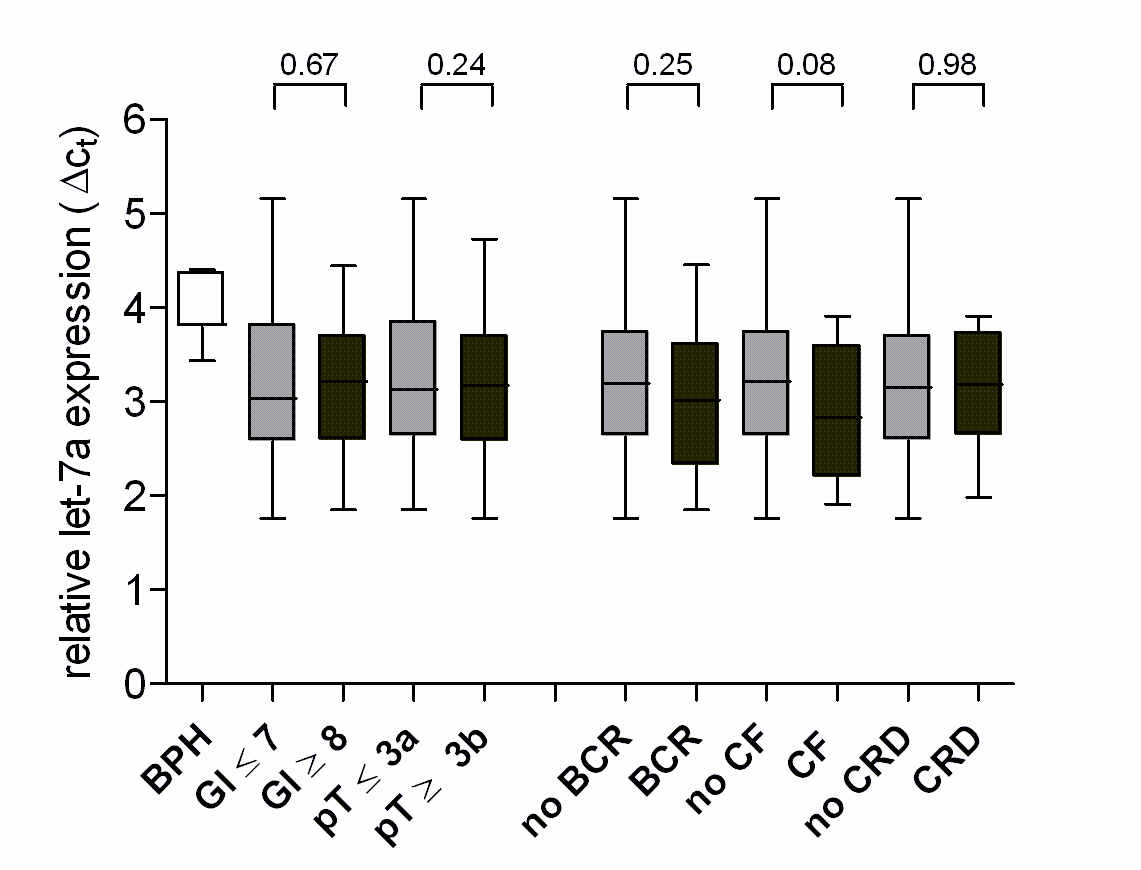

Supplement: Figure S2 — Association of let-7a expression and clinical data. Box- and whisker-plot show expression of let-7a in 98 high-risk PCa tissue and 6 BPH specimens; assessed by qRT-PCR. Subgroups are based on: • pathologic tumor features like Gleason Score and pathological tumor stage (GS ≤7, pT ≤3a–light grey), (GS ≥8, pT ≥3b–dark grey) and • clinic-pathological characteristics like BCR, CF, CRD (no BCR/no CF/no CRD–light grey), (BCR/CF/CRD–dark grey). Let-7a expression is not associated to clinical data in high-risk PCa. Expression is shown as means with error bars for standard deviation. p values were calculated using the Welch 2 sample t-test. (TIFF) [file pone.0065064.s002.tiff]

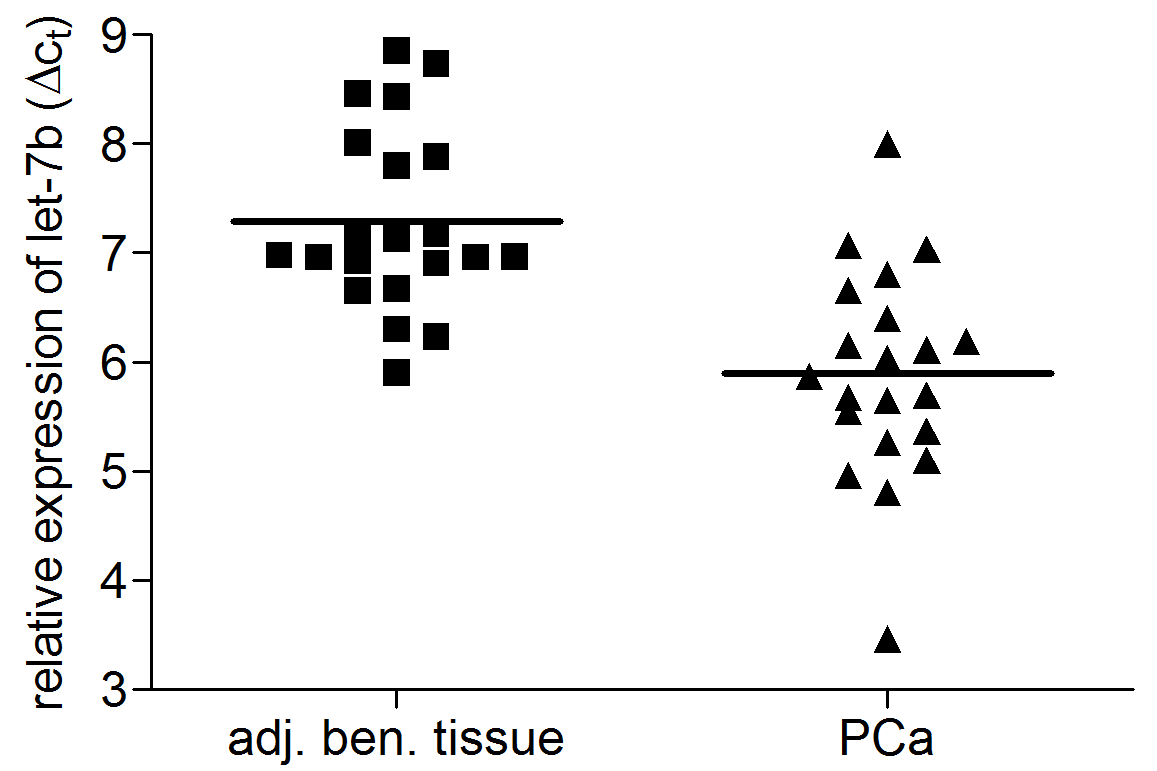

Supplement: Figure S3 — Let-7b expression in cancerous and adjacent benign prostatic tissue (cohort C). Scatter Plot shows expression of let-7b in 21 pairs of PCa tissue and adjacent benign tissue. Median expression of let-7b is significantly lower in the cancerous compared to the benign tissue. p values were calculated using the Welch 2 sample t-test. (TIFF) [file pone.0065064.s003.tiff]
